# Women's reluctance for pregnancy: Experiences and perceptions of Zika virus in Medellin, Colombia

**DOI:** 10.1002/ijgo.13046

**Published:** 2020-01-23

**Authors:** Veronika Tirado, Santiago A. Morales Mesa, John Kinsman, Anna Mia Ekström, Berta N. Restrepo Jaramillo

**Affiliations:** ^1^ Department of Global Public Health Karolinska Institutet Stockholm Sweden; ^2^ Faculty of Psychology Institute of Health Sciences (CES University), University Medellín Colombia; ^3^ Faculty of Law and Political Sciences Catholic University Luis Amigó Medellín Colombia; ^4^ Department of Epidemiology and Global Health Umeå University Umeå Sweden; ^5^ Department of Infectious Diseases Karolinska University Hospital Stockholm Sweden; ^6^ Department of Virology Colombian Institute of Tropical Medicine – Institute of Health Sciences (CES University) Medellín Colombia

**Keywords:** Colombia, Family planning, Perceptions, Pregnancy, Zika virus

## Abstract

**Objective:**

To explore how being infected with the Zika virus during pregnancy was experienced by affected women, and how it influenced their family relationships and future family planning.

**Methods:**

We conducted a qualitative study, including 19 semistructured interviews with women of reproductive age and confirmed Zika infection during 2015–2018 in Medellin, Colombia. Purposeful sampling was applied, and participants were identified through National Public Health Surveillance System records. Interviews were recorded, transcribed verbatim, and analyzed using content analysis with inductive and deductive approaches.

**Results:**

Of 19 women interviewed, eight women identified the pregnancy as unexpected and two women had undergone permanent sterilization. Women had mixed views about decision‐making related to family planning, and not having an abortion in a future pregnancy was influenced by religious beliefs. Women knew about vector‐borne transmission but were not well informed about sexual transmission of the virus. Women desired better support and guidance to ease concerns about Zika virus.

**Conclusion:**

All interviewed women expressed a need for more information about Zika virus and continuous support, specifically after delivery, from healthcare professionals. Communication strategies to enhance culturally sensitive messages and for accurate perception of information are recommended during Zika outbreaks.

## Abstract 2

Women interviewed were unaware of sexual transmission of the Zika virus. Women desired continuous counselling and support during their pregnancy and after the birth.

## 1 INTRODUCTION

Zika virus is a major concern for women of reproductive age in endemic settings given its link to serious birth defects in infants born to mothers infected with the virus during pregnancy. The susceptibility of becoming infected with Zika virus appears to be similar in pregnant compared with nonpregnant women, and up to 80% of infected individuals are asymptomatic, while the rest experience fever, arthralgia, conjunctivitis, and maculopapular rash, similar to chikungunya and dengue infection.^1^, ^2^ The most common adverse pregnancy outcomes associated with Zika virus, known as congenital Zika syndrome (CZS), include microcephaly, brain atrophy, and intracranial calcification in the fetus.^2^, ^3^ Microcephaly is a birth defect where the baby has a smaller head compared with other babies of the same age and sex. CZS is not limited to brain atrophy and asymmetry; it may also include abnormally formed or absent brain structures, eye abnormalities such as retinal lesions, as well as other anomalies such as excessive and redundant scalp skin.^4^

The Zika outbreak in Colombia officially began in October 2015 when nine cases were confirmed by laboratory tests.^5^ The number of incident cases peaked in February 2016 when more than 6000 cases per week were reported in Colombia.^6^, ^7^ With over 100 000 confirmed or suspected cases of Zika virus, Colombia has had the second largest number of reported cases in the world, after Brazil. Between 2016 and 2018, nearly 400 children were diagnosed with microcephaly in the country,^8^, ^9^ but there may be more cases of CZS that are unaccounted for in Colombia's surveillance system.

Zika virus is transmitted predominantly through the bite of infected *Aedes aegypti* mosquitoes, which also transmit dengue, chikungunya, and yellow fever.^10^ Community programs for disease prevention in Colombia have been focusing primarily on mosquito control, such as to eradicate or control mosquito breeding areas.^11^ However, Zika virus can also be transmitted during pregnancy from a woman to her fetus, and through unprotected sexual activity.^12^ A study in Brazil found high general awareness of the Zika virus, while women's knowledge about sexual transmission was low.^13^ The fact that this mosquito‐borne virus can also be transmitted through sexual contact makes it a unique public health challenge, adding to the risk of Zika infection among women.

The Zika virus outbreak highlighted barriers to sexual and reproductive health and rights (SRHR) in Latin American countries, such as Brazil and Colombia, including restrictive sexual and reproductive health (SRH) policies and reduced access to SRH services. In 2006, Colombia decriminalized abortion only under exceptional circumstances, including life‐threatening maternal conditions and severe fetal brain malformations, such as those seen in CZS.^14^ The cost of different family planning methods can act as an economic burden and access to SRH services is a challenge in Colombia; particularly for women on low incomes living in remote and rural areas owing to lengthy travel distances to clinics.^6^ Previous research in Brazil has identified health system shortcomings in adequately protecting children with disabilities associated with CZS, and that conservative gender norms and gender inequalities seriously limit access to SRH services.^15^

To improve prevention and management of Zika virus infection, the aim of the present study was to understand the perceptions among women of reproductive age who had been infected with Zika, the impact on their families, and their reproductive choices and plans.

## 2 MATERIALS AND METHODS

A qualitative approach using content analysis was considered appropriate for our study to understand women's experience of being infected with Zika virus during pregnancy, concerns about CZS, and perceptions on family planning.

The study was conducted in Medellin, Colombia—a city of over 2.8 million inhabitants in 2017, of whom an estimated 54.4% are women.^9^ Study participants were identified through the National Public Health Surveillance System (SIVIGILA) records from October 2015 to April 2018 with the support and collaboration of the Secretariat of Health in Medellin. We opted for purposeful selection sampling from SIVIGILA to identify and select women who were of reproductive age, had had live births, and resided in the city of Medellin.

Inclusion criteria for participants were: (1) age 18–45 years; (2) diagnosis of Zika virus during pregnancy; (3) permanent resident and residing in Medellin; (4) Zika virus infection reported to the National Institute of Health in Colombia and the infant considered a suspected case of CZS (i.e. confirmed by clinical or laboratory diagnosis by reverse transcriptase polymerase chain reaction [PCR] test and/or the following symptoms: fever, red eyes without discharge, skin rash, arthralgias, headache, lumbago, neurological and immunological affections or congenital abnormalities)^16^; (5) live birth; and (6) able and willing to give informed consent as well as understand and comply with the study. We excluded women who were travelers/visitors not from Colombia and non‐Spanish speakers, as well as those with reported co‐infections. During October 2015 to April 2018, a total of 297 women infected with Zika virus—including 113 pregnant women—were reported to SIVIGILA. Nineteen women who met the inclusion criteria were invited to participate in the interview, and all of them agreed.

After establishing formal permission to conduct the study from the Secretariat of Health in Medellin, informed consent was provided over the telephone from women who were registered as a confirmed case of Zika infection and whose baby was suspected to have CZS. We informed the potential participants about the study objectives and goals, the voluntary aspects of participating in the interview, potential risks and measures taken to prevent those risks, as well as the mode of the participation for the face‐to‐face interview. After we had obtained written consent, the participants were still able to withdraw at any point during the face‐to‐face interview without any negative consequences for their healthcare services.

Participants were asked if they preferred a male or a female interviewer and if there was a preference in the location of the interview, as we wanted them to feel comfortable. To facilitate attendance, we provided reimbursements for any transportation costs. Semistructured interview was chosen as the method of data collection. The research team constructed and piloted an interview guide with questions in Spanish, probing areas under topics related to awareness of Zika virus and means of information, experiences during the pregnancy and family planning in relation to abortion, as well as experiences surrounding healthcare staff and core support system (supporting information Data S1). The interviewers considered the nature of sensitive topics during the interviews and allowed the participants adequate time to reflect and respond, and they were also ready to clarify any points of uncertainty about Zika as necessary. All interviews were performed in Spanish by either VT or SAMM and BNRJ, who observed each of the interviewees’ reactions and gestures. Interview duration was between 40 and 60 minutes. Interviews were conducted until saturation was reached and further data collection did not contribute to new information on the main topics.

The interviews were recorded and transcribed verbatim, and only the research team had access to the data. The researchers preserved the anonymity and confidentiality of the women who were interviewed, and conducted the data analysis with replaced names, along with all identifiable information. In accordance with Graneheim and Lundman,^17^ data analysis was processed through a systematic classification process of coding and identifying meaning units, defined as words or sentences. The subsequent codes, subcategories, and categories were developed by both inductive and deductive approaches using manifest content‐analysis mapping to validate the original categories in Spanish utilizing ATLAS.ti version 7.5.2 (ATLAS.ti Scientific Software Development GmbH, Berlin, Germany) (supporting information Figure S1). The deductive approach allowed us to emphasize the general subcategories, while the inductive approach focused on new codes and meaning units. The final categories were generated using an inductive approach in English to consolidate the original categories using Excel (Microsoft; Redmond WA, USA). An example of a trail analysis is shown in supporting information Figure S2.

We considered several aspects to improve the trustworthiness of our research. The researchers recognized the possibility of respondent bias and this might have resulted in biased interpretations. Parts of the interview material were initially coded by SAMM and then compared by VT ensuring codes were derived from the raw data in an attempt to mitigate researcher bias. If discrepancies were encountered, they were discussed within the research team, and the differences in coding were discussed until the interpretation could be agreed by all. Revisions were conducted on the primary data and during the analysis and writing process by continuous discussions among the research team. Other ethical principles, such as confidentiality and anonymity of research participant, were applied.

The study was approved by the Colombian Institute of Tropical Medicine Ethics Committee and PAHO ERC No. 2017.07‐0070.

## 3 RESULTS

We conducted 19 semistructured interviews with women diagnosed with the Zika virus during pregnancy, including three women who had children with adverse physical outcomes, including microcephaly, brain atrophy, and arthrogryposis. There were no follow‐up interviews. The sociodemographic characteristics of the participants are shown in Table 1. The women reported that their diagnosis of Zika virus had occurred mainly during the third trimester of pregnancy (from week 27 to the end of the pregnancy). All 19 women had given birth to a child that was suspected to have CZS. The ages of the women varied between 18 and 38 years. Six women were single and two women were separated, while eight had long‐term partners and three were married. All interviews were conducted in Medellin, Colombia.

**Table 1:** Sociodemographic characteristics of the 19 women interviewed

Characteristics	No.
Age, y	
18–24	8
25–31	5
32–38	6
Relationship status	
Single	6
Married	3
Cohabiting/living together	8
Other (separated/divorced)	2
Educational level	
Higher/university	4
Higher/non‐university	6
Secondary education	8
Primary education	1
Socioeconomic status^a^	
Lower‐class (*estrato* 1–2)	14
Middle‐class (*estrato* 3–4)	5
Occupation	
Employed	9
Unemployed	9
Student (not employed)	1

a Socioeconomic status defined as *estrato* in Colombia. The *estrato* number is designated to an individual depending on household income and residence zone. People living in *estrato* 1 are considered lowest class; *estrato* 2: low‐middle class; *estrato* 3: middle class; *estrato* 4: upper‐middle class; *estrato* 5: upper class; and *estrato* 6: wealthy. Source: National Statistic System (DANE). http://www.dane.gov.co/index.php/69-espanol/geoestadistica/estratificacion/468-estratificacion-socioeconomica. Accessed June 3, 2019.

All 19 participants expressed their perceptions and provided their experiences of having been infected with Zika virus. During the analysis process, subcategories were grouped into clusters that generated similar concepts consistent with each participant's experiences and perceptions. The identified categories were: knowledge of Zika virus; support at the time of diagnosis; concerns about Zika virus; uncertainty over a new pregnancy and childbearing; and accuracy of the information about Zika virus. Categories and subcategories are shown in Table 2.

**Table 2:** Overview of the themes, categories, and subcategories

Categories	Knowledge about Zika virus	Support at the time of diagnosis	Concerns about Zika virus	Uncertainty over a new pregnancy and childbearing	Accuracy of the information about Zika virus
Subcategories	Heard about Zika virus and where to find information	Reliance on the partner and family	Meeting other people with Zika virus	Avoidance of (or intention to avoid) becoming pregnant	Informed about another diagnosis *(*not Zika virus*)*
	Informed about ways to prevent transmission by mosquitoes	Changes in the relationship with partner	Feeling worried about consequences at the time of diagnosis	Contemplated a decision about abortion	Zika virus test result was not received
	Informed about the consequences related to Zika virus *(*microcephaly)	Reliance on healthcare professionals	Experiencing emotions about the baby with microcephaly	Incomplete information about sexual transmission of Zika virus
	Aware of the differences between Zika virus, dengue, and chikungunya	Services provided by healthcare professionals	Unaware of and misconceptions about Zika virus

### 3.1 Knowledge about Zika virus

All 19 women had previously heard about Zika virus and knew where to find additional information. The women mentioned several sources of information about the virus, such as television or radio, from healthcare professionals or doctors, and attending workshops in their community or school seminars. One participant described:

Because you never think that it is going to happen to you, then you never pay attention to it. I did watch it (on television), but when we were in school, they told us we were going to have a class about that, but I didn't remember all of it. I remembered the symptoms. I remember watching all that and saying, oh my God, this is what's going on. Participant 8, age 21

Participants mentioned that the information shown on television or heard on radio had a greater impact not only to become cautious about contracting Zika virus, but also learning about the different types of mosquitoes. A participant said:

Well, to tell you the truth, when I watched it on television, I didn't believe that could happen to me, there have been many types of mosquitoes, and that never happened. When they told me that I [had it], I didn't believe it because I thought it usually only happens on television, until I had to live it. I have this experience now. Participant 17, age 30

Regarding prevention of Zika virus infection, all of the women were aware of different ways to prevent becoming infected with Zika virus by mosquito bites. For instance, preventing becoming bitten by mosquitoes was exemplified by the participants. One woman said:

I can't be with long sleeves and long pants every day. The mosquito can bite me here or even in the tip of my finger. That is very hard to control. Participant 5, age 20

Participants also reported means of preventing the spread of Zika virus by eliminating the breeding sites for the mosquito. The women knew about the possible consequences during pregnancy when Zika virus occurred; this was sometimes referred to as microcephaly, but mainly described as malformations to the baby's head. One of the participants stated:

We learned that it was a mosquito that bit pregnant women, and that made the baby born with malformation. Participant 7, age 23

When asked about the differences between Zika virus and other mosquito‐borne diseases, including chikungunya, one of the participants stated:

I have heard about chikungunya and Zika, but I heard it was almost the same thing, the same results. Participant 1, age 20

The women indicated that they were familiar with the different mosquito‐borne diseases, and the true impact of dengue or chikungunya was better understood when people in their communities or close friends had experienced such disease.

### 3.2 Support at the time of diagnosis

All 19 participants reflected on the type of support given at the time of diagnosis. Four subcategories emerged within this theme, including reliance on their partners and families, changes in their relationship with their partner, reliance on healthcare professionals, and health services. Most of the women received some form of financial support from her partner and family. Regarding care of the child, participants stated the possibility of leaving their child under the care of the extended family as an element that contributed to the quality of life of the family. When asked about the relationship with their partner after Zika virus diagnosis, many of the participants mentioned that there was significant change in the interaction between the father and their child. As described by one participant:

During the pregnancy, the father of my baby provided me with everything I needed and for my baby. Now, however, we are not living together. Participant 19, age 33

A key aspect in terms of support provided to the women, with an influence on the quality of life of the child, is determined by the role of healthcare professionals. The participants positively assessed the possibility of sharing their doubts and concerns with healthcare professionals. However, their experiences concerning the type of healthcare support was contradictory; for instance, one of the participants expressed feeling unfairly judged when she had visited the doctor. The words of this mother illustrate this:

After seeing how the doctor behaves with my other daughter, I noticed a doctor discriminates her [the baby] a lot and wanted me to feel bad for my daughter's medical condition. Participant 11, age 36

After they had been diagnosed, participants expressed the need for better support, guidance, information about Zika virus, and further advice from healthcare staff, particularly after giving birth.

The last 15 days of my pregnancy the medical care was very good, but after she [the baby] was born, nothing is authorized, and I am sent to places where they tell me they don't have a healthcare specialist for her care. Participant 18, age 38

Even though most of the information about Zika virus was provided by the local radio news, several women mentioned that they would have preferred to be invited to an educational session with healthcare personnel during their pregnancy to talk more about microcephaly and other worries in relation to Zika virus. One participant mentioned:

The information on television was helpful, but I wanted to know more about the situation with Zika, so that you can take more preventive measures than what they say in the news. Participant 1, age 20

### 3.3 Concerns about Zika virus

The immediate emotional response mentioned by the participants was concern about the possible Zika‐related health complications during pregnancy, specifically for the fetus. Concerns about Zika virus were expressed in three subcategories: meeting other people with Zika virus, feeling worried about consequences at the time of diagnosis, and experiencing emotions about having a baby with microcephaly. All 19 women experienced feeling worried about their baby at the time of diagnosis, and other types of emotions were referred to as concerns and fear of the baby being born with microcephaly. When asked about their concerns surrounding babies born with microcephaly, one mother said:

It was very difficult, very hard. Aside from the pregnancy, I would not worry about myself, but only for him [the baby]. Participant 4, age 31

Another mother mentioned:

I got scared when I was told my baby could come out with problems like microcephaly or brain paralysis or many other things – I imagined my baby was going to be born with all that. Participant 17, age 30

The participants expressed that their concerns about Zika virus had not changed as the pregnancy progressed. All 19 participants summarized their fears of having a child diagnosed with microcephaly.

Women who had met someone infected with Zika virus also said that they became more worried about their prognosis at the time of pregnancy. The women were also disturbed by the images of newborns with microcephaly. One mother said:

I tried not to read stuff about Zika, because it would have been more traumatizing to see what I saw after I had her. It was better not to see that, otherwise the pregnancy would have been more difficult. Participant 5, age 20

When asked about social interactions with other people who had had the Zika virus, participants mentioned potential social stigma. One mother said:

I think no one is prepared to see children with microcephaly, because there are mothers who don't accept it and have difficulties to work – I think people are not prepared to work for the children affected with microcephaly. Participant 18, age 38

### 3.4 Uncertainty over a new pregnancy and childbearing

Eight of the participants (42%) identified the pregnancy as unexpected/not planned, and two participants (10%) had undergone permanent sterilization to prevent a new pregnancy. When asked if the women were planning to have more children, many of the respondents expressed both desire and unwillingness to become pregnant again. One woman said:

[I don't want any more children]… because she [the baby] needs a lot of care and I have to pay a lot of attention to her. I could also have another baby with microcephaly, so No. Participant 19, age 33

While another participant said:

I don't know if I want another baby, I would let God decide that. Participant 4, age 31

Despite the mother's diagnosis of Zika virus at the time of pregnancy, not all children had microcephaly. Nevertheless, the participants had contemplated a decision about abortion in a future pregnancy, which was also influenced by healthcare professionals.

I thought about it… if the doctors tell me that my baby was going to be born with malformations or with Down syndrome, and they gave me the opportunity to interrupt the pregnancy, I maybe would have done it. From a medical point of view, it's a disease and I would think about it, but from an emotional point of view, I can't consider deciding against my baby's life. Participant 5, age 20

Another mother explained that contemplating the decision would rely on the care of her other children, such as:

If the doctor tells me that the pregnancy might put me in danger, I would also think about it – I have three other girls and if the baby is coming sick, knowing that whatever God gives you is for a reason, I would… yes, it sounds difficult. Participant 17, age 30

When asked about the hypothetical situation of becoming pregnant again during another outbreak of Zika virus, all 19 mothers responded that they would continue the pregnancy based on personal or religious beliefs. One participant said:

It would be very difficult for me… I would have him [the baby], eventually. It is still a life; he is not guilty of anything. Participant 18, age 38

Most of the women sought prayer as support during pregnancy. The baby was not seen as responsible for the mother's decision to continue the pregnancy. One woman said:

I would take the risk, completely. He [the baby] is not guilty of anything and I would risk having him. If in case he's born with problems, that is God's will, but if God gives him to me and as long as I am able to have him, I would risk it no matter the difficulty. Participant 19, age 33

### 3.5 Accuracy of information received about Zika virus

Information provided by different media sources and healthcare professionals about Zika virus was perceived as inaccurate by the participants. Despite being a registered case of Zika virus, many of the women were given another diagnosis other than Zika virus, which resulted in a late or unclear diagnosis. One woman said:

I had Zika and chikungunya tests, but at the time, it was also dengue… I did not know I had Zika, they said I probably had dengue and they would call, but they never called me. Participant 4, age 31

The participants were knowledgeable and willing to be informed about prevention of Zika virus and mosquito‐borne transmission. The women were asked to share their understanding of how Zika virus is transmitted, and all 19 knew that it is transmitted by mosquitoes and it affects people, especially women who are pregnant. However, when the women were asked if they had heard that Zika virus can be transmitted through sexual contact, all 19 participants said they did not know.

Hence, many women were surprised when asked what they had heard about Zika virus and its modes of transmission. One participant said:

I never heard that Zika could be transmitted through sexual contact, but it makes sense, because if that is in the blood then any kind of contact, it can also happen… then the media should also say that was [sexual transmission] if it is true about Zika. Participant 3, age 18

There were also misconceptions about Zika virus in relation to transmission only occurring in warmer climates. One of the participants said:

God, in what moment did that mosquito bite me, I was always in places where it was cold. I was pregnant, and so the mosquito must have gone in my house where there was dirt. Participant 7, age 23

## 4 DISCUSSION

This qualitative study explored experiences and perceptions of Zika virus during pregnancy among 19 women in Medellin, Colombia. The participants were registered as cases of Zika virus in SIVIGILA that had been confirmed by healthcare staff on the basis of clinical symptoms or laboratory diagnosis made by reverse transcriptase PCR test. However, the majority of the women felt that their diagnosis was unclear. In addition, laboratory confirmation of Zika virus is challenging owing to the possible cross‐reactivity with dengue,^18^ possibly leading to a misdiagnosis. Therefore, providing information about potential co‐infections of Zika virus and other aspects of the diagnosis for people at risk is essential to counter any misconceptions diffused through mass media and different communication channels that may lead to missing information about the virus.

All participants knew that the primary source of information was from the health authorities. The women independently sought more details about Zika virus from the local news coverage, such as the television or radio, and by accessing the internet. However, the women interviewed were not well informed about all transmission modes. Participants did not refer to condom use as a form of prevention against Zika virus, and this is explained by the participants’ low knowledge about sexual transmission. Other qualitative studies have indicated a high awareness of CZS among women of childbearing age at risk of Zika virus; however, little information was provided to them about sexual transmission.^19^, ^20^ Our findings seem to corroborate and support this perception.

The interviews with the women revealed that cases of Zika virus were more common among young and unemployed women of low and middle socioeconomic status. Lack of proper infrastructure and poverty may lead to disparities in service provision and information. We found that all 19 participants had access to reproductive healthcare services. A recent study on Zika virus and microcephaly in Brazil found that almost all registered cases were from low‐income families, suggesting that the epidemic both reflects and may widen socioeconomic inequalities. For example, women in Brazil with a child born with Zika‐related health complications tend to be young, single mothers in relative poverty, living in remote parts of the country.^21^ These factors increase the risk of disease transmission and the economic burden on the most vulnerable populations.

All 19 women were the prominent caregivers to their babies. We found that the participants were reliant on either their partners and family or on healthcare services overall. Thus, individual quality of life and family dynamics are impacted in many ways; for instance, previous research highlighted several challenges families of children with CZS may face, such as complex medical care and economic burden, uncertainty for children about the unfolding consequences of CZS, limited professional knowledge, and potential social isolation and stigma.^22^ Male involvement and their role in SRH are essential to consider for the care of children affected by Zika virus, especially in patriarchal societies.

Women reported that support from healthcare providers for care of their children outweighed the immediate social and family support. Many of our participants were relieved to be able to share their doubts and concerns with healthcare professionals throughout their pregnancy. However, it was evident that the participants diagnosed with Zika virus desired further support from healthcare professionals, particularly after giving birth, to comprehend and gain more information on unexpected consequences related to Zika virus. Despite the existence of universal health coverage and an integrated social welfare support system in Colombia, no long‐term programs have been established to specifically support children born with Zika‐related health complications.^6^

The women did not report signs of mental health disorders. However, much of the necessary healthcare support described by the participants, such as providing accurate information and supportive communication, may also apply to other psychosocial support including management of mental disorders and strengthening social support and social networks. It is important to note that information for and referrals to specialized healthcare support services, such as psychological support or counseling, were provided to all of the women interviewed as part of the study protocol, and at least one of the participants sought counselling immediately after the interview.

Colombia remains among the countries with the highest number of Zika virus cases, with 182 cases reported in 2019, as of May 31.^7^ Although the impact and consequences of CZS remain uncertain for future pregnancies, postponing pregnancy or contemplation of abortion was suggested as possibilities by the women interviewed. The participants presented divided views in relation to having an abortion for a future pregnancy. Since abortion remains legal in Colombia in the case of rape, incest, or to protect a woman's health, some women agreed that they would proceed with an abortion if it was recommended by medical professionals. However, personal or religious beliefs and practices as reasons not to have an abortion were expressed by all of the participants when presented with a hypothetical scenario of another pregnancy and another Zika virus outbreak. Research in Brazil explored the religious influences on society and political aspects in relation to highly stigmatized infections such as HIV and AIDS.^23^ A possible explanation could be that religious beliefs are still highly influential on social attitudes and public policy in Latin America.

More subtle but still significant is the impact of the disease during pregnancy and the unknown long‐term effects of the virus, which seem to influence decision‐making on family planning. SRHR regulations in Colombia aim to prevent unwanted pregnancies, and do not require parental consent for SRH counseling or access to contraception.^24^ We found that some of the women interviewed desired a future pregnancy, but others expressed unwillingness for another pregnancy and childbearing. Some of the participants also reported the use of long‐term contraceptive methods, and two women had been sterilized, reflecting their clear intention to avoid unwanted pregnancies. It is important to combat the Zika virus, and equally important to educate and provide access to SRH services and specialized care to women who may be emotionally traumatized by the uncertainty of exposure to Zika virus.

Many of the women interviewed perceived the prevention of Zika virus transmission during their pregnancy as their sole responsibility, in part because the health authorities had failed to communicate clear information about sexual transmission of the virus. SRHR remains an essential component during the outbreak of Zika virus in Colombia and making an informed decision for family planning is crucial, both for couples who plan to get pregnant or to delay it, and for those who are already pregnant.

In conclusion, this study explored the experiences and perceptions of Zika virus among women during their pregnancy. Further research is recommended to explore the impact of Zika virus on the uptake of family planning services, unmet needs from these services, and the role of male involvement in women's sexual and reproductive health. In addition, the sources of information, such as health authorities and governmental sources, should work to ensure provision of clear and unambiguous recommendations and advice that is culturally sensitive to stress the key messages in the context of Zika virus.

## AUTHOR CONTRIBUTIONS

All authors contributed to the planning and proposal of the research project. VT, SAMM, and BNRJ conducted the data collection, analysis, and writing of the original report. All authors contributed to reviewing drafts of the paper. All authors read and approved the final manuscript.

## CONFLICTS OF INTEREST

The authors have no conflicts of interest.

## Supporting information


**Data S1.** Interview guide (Spanish/English).
**Figure S1.** Mapping of the categories and subcategories developed using both inductive and deductive approaches (Spanish).
**Figure S2.** Example of a trail analysis.
