# Supplementary material for: Women's reluctance for pregnancy: Experiences and perceptions of Zika virus in Medellin, Colombia
Source: Int J Gynaecol Obstet. 2020 Jan 23;148(Suppl 2):36–44. doi: 10.1002/ijgo.13046 (PMC7064904; doi:10.1002/ijgo.13046)
Supplement: Supplementary file 1 — Data S1. Interview guide (Spanish/English). Figure S1. Mapping of the categories and subcategories developed using both inductive and deductive approaches (Spanish). Figure S2. Example of a trail analysis. [file IJGO-148-36-s001.docx]

**Supporting information S1.** Interview guide (Spanish/English)

**Conocimientos sobre Zika / *Knowledge about Zika virus***

1. ¿Podría decirme usted qué sabe sobre el virus Zika?

*Could you tell me what you know about Zika virus?*

- - Que aspectos del virus Zika le gustaría saber más?

*What aspects of the virus would you like to know more about?*

**Fuentes de información sobre Zika / *Sources of information about Zika virus***

1. Dónde has recibido la información sobre Zika? (medios sociales, televisión, servicios de salud)

*Where have you received the information about Zika (social media, television, health services)*

- - Cúales de las fuentes de información crees que ha sido mejores y que han sido las menos útiles?

*Which of the sources of information do you think have been the best or least useful?*

**Experiencia sobre Zika / *Experience with Zika virus***

1. Alguién de su familia ha sido infectado por Zika y cómo ha sido esa experiencia?

*Has anyone in your family been infected by Zika virus and how has that experience been?*

- - Usted ha sido infectado por Zika. Recuerda cúando fue/cómo fue su experiencia?

*When you were infected with Zika virus, how was your experience?*

- - ¿Cómo supo que estaba infectado por Zika?

*How did you find out you were infected by Zika virus?*

1. Cómo fue el apoyo emocional/social durante el embarazo y Zika de su:

*How was the emotional/social support during pregnancy from your:*

- - Pareja, Familiares y/o Personas en el barrio comunidad?

*Partner, family, and/or people in your neighborhood?*

- - ¿Ha cambiado su relación con su pareja por esta enfermedad?

*How has the relationship with your partner changed because of the diagnosis?*

1. Ha buscado alguna institución de salud?

*Have you visited any health institution?*

- - ¿Le hicieron una prueba para confirmar Zika?

*Can you describe how the process was when you tested for Zika virus*

- - ¿Cómo fue la atención o servicios médicos (cuáles fueron las recomendaciones y qué les hizo falta, alguna información)?

*How was the medical care or services (What was the recommendation?)*

**Embarazo, posponer embarazo, aborto / *Pregnancy, postpone pregnancy, abortion***

1. Tiene hijos y/o está embarazada ahora?

*Do you have children and/or are you pregnant now?*

- - Cuántos hijos tiene?

*How many children do you have?*

- - ¿Cómo ha sido la reacción de los niños a un bebé con microcefalia?

*How was the reaction of your children to the baby with microcephaly?*

1. Está planeando quedar embarazada otra vez?

*Are you planning to get pregnant again?*

- - ¿Si es así, está usted haciendo algo para evitar ser infectado por Zika?

*If so, are you doing anything to avoid being infected by Zika virus?*

1. Sabe usted de otras mujeres en su comunidad/barrio que han querido posponer el embarazo como un medio para evitar un bebé con microcefalia?

*Do you know of other women in your community/neighborhood who have wanted to postpone pregnancy as a means to avoid a baby with microcephaly?*

- - Si es así, ¿que han dicho las personas acerca de esto?

*If so, what have people said about this?*

1. Qué sabe usted de la transmissión de Zika por relaciones sexual (fluidos)?

*What do you know about transmission of Zika virus by sexual relations?*

- - ¿Qué métodos utiliza durante la relación sexual para prevenir Zika?

*What methods do you use during sex to prevent Zika virus?*

1. ¿Si usted se enfermara (otra vez) de Zika durante el embarazo y supiera que su bebé tiene algún problema congénito por esta infección (ej. microcefalia por Zika), Usted optaría por el aborto? Si no quiere responder esta pregunta no hay problema

*If you become ill (again) with Zika virus during pregnancy and knew that your baby has a congenital problem due to this infection (e.g. microcephaly due to Zika virus), would you opt for abortion? If you do not want to answer this question, you do not have to.*

**Preguntas finales / *Final questions***

1. ¿Qué le preocupa el Zika?

*What worries you about Zika virus?*

1. ¿Alguna vez ha sido infectado con dengue o Chikungunya?

*Have you ever been infected with dengue or chikungunya?*

- - ¿Crees que puedes decirme la diferencia entre Zika, dengue Chikungunya?

*Do you know the differences between Zika virus, dengue, and chikungunya?*

**Supporting information S2**. Mapping of the categories and subcategories developed using both inductive and deductive approaches (Spanish).


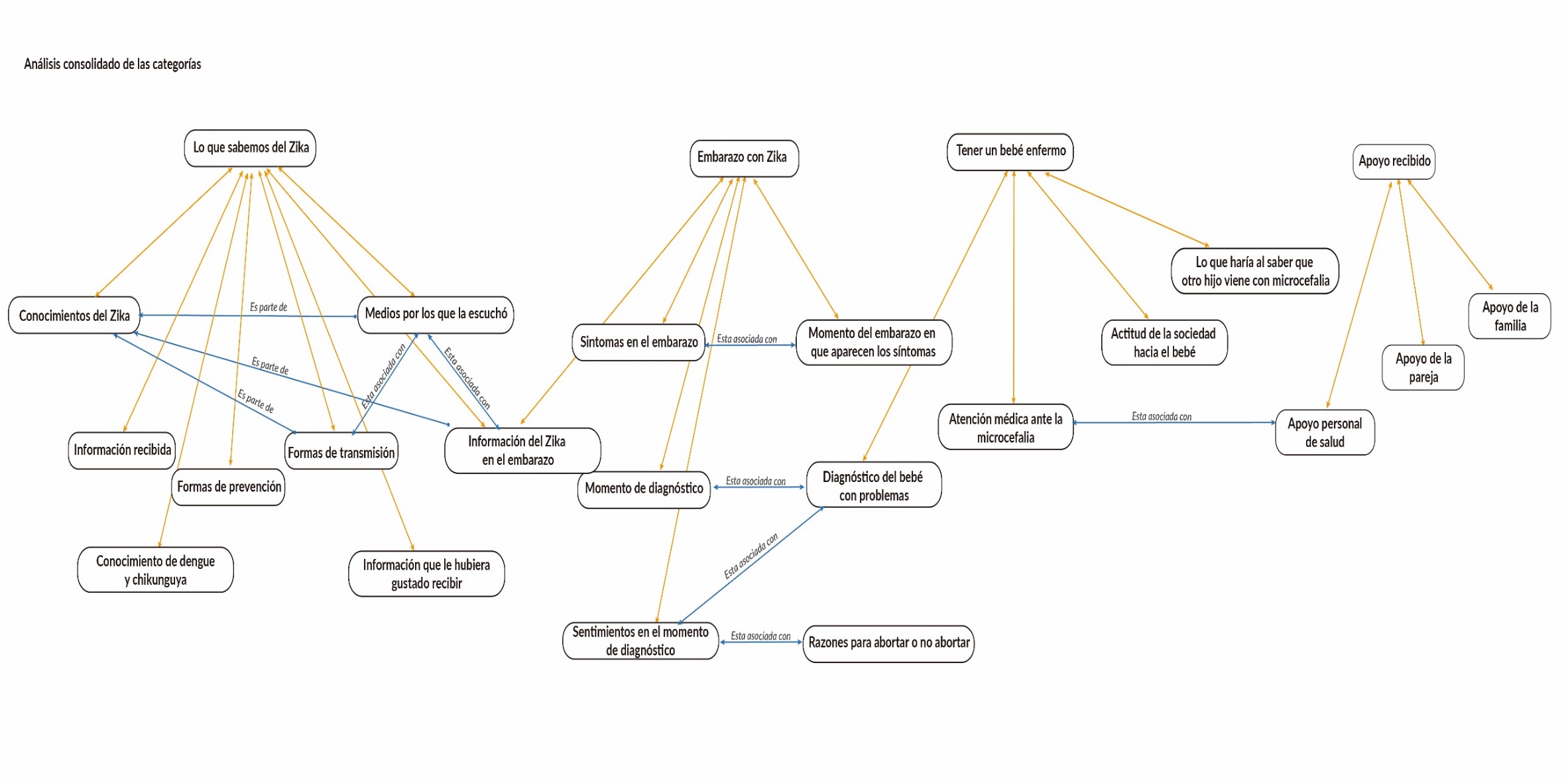


*The consolidated analysis and categories in English: What is known about Zika virus - *Lo que sabemos del Zika;* Knowledge-base about Zika virus - *Conocimientos del Zika*; Received information - *Información recibida*; Knowledge about dengue and chikungunya - *Conocimiento de dengue y chikungunya*; Ways to prevent - *Formas de prevención*; Transmission modes - *Formas de transmisión*; Information to would have liked to receive - *Información que le hubiera gustado recibir*; Sources of information - *Medios por los que la escuchó*; Information about Zika virus during pregnancy - *Información del Zika en el embarazo*; Pregnancy with Zika virus *- Embarazo con Zika*; Symptoms during pregnancy *- Sintomas en el embarazo*; At the time of diagnosis *- Momento de diagnóstico*; Feelings at the time of diagnosis *- Sentimientos en el momento de diagnóstico*; Reasons to abort or not abort *- Razones para abortar o no abortar*; Diagnosis of an ill baby (microcephaly) *- Diagnóstico del bebé con problemas (microcefalia)*; Time of pregnancy when symptoms appear *- Momento del embarazo en que aparecen los síntomas*; Having an ill baby (microcephaly) *- Tener un bebé enfermo (microcefalia)*; Medical care for microcephaly *- Atención médica ante la microcefalia*; Societal attitudes towards the baby *- Actitud de la sociedad hacia el bebé*; Reaction to having another child with microcephaly *- Lo que haría al saber que otro hijo viene con microcefalia*; The support received *- Apoyo recibido*; Support from healthcare staff *- Apoyo personal de salud*; Partner support *- Apoyo de la pareja*; Family support *- Apoyo de la familia;*

**Supporting information S3.** Example of a trail analysis.

**Core:**

Interviewer 1: “Do you think you received more attention because of the Zika diagnosis?”

Respondent: “No, I mean, they attended to me well, thank God.”

**Category:**

Support at the time of diagnosis

**Condensed – code:**

Healthcare providers - services

**Subcategory:**

Services provided by healthcare professionals

**Theme:**

Comprehension
